# Copulatory courtship by internal genitalia in bushcrickets

**DOI:** 10.1038/srep42345

**Published:** 2017-02-07

**Authors:** Nadja C. Wulff, Thomas van de Kamp, Tomy dos Santos Rolo, Tilo Baumbach, Gerlind U. C. Lehmann

**Affiliations:** 1 Humboldt University Berlin, Department of Biology, Behavioral Physiology, Invalidenstrasse 43, 10115 Berlin, Germany; 2 Karlsruhe Institute of Technology (KIT), Laboratory for Applications of Synchrotron Radiation (LAS), Kaiserstr. 12, 76131 Karlsruhe, Germany; 3 Karlsruhe Institute of Technology (KIT), Institute for Photon Science and Synchrotron Radiation (IPS), Hermann-von-Helmholtz-Platz 1, 76344 Eggenstein-Leopoldshafen, Germany

## Abstract

Male genital organs are among the fastest evolving morphological structures. However, large parts of the male’s genitalia are often hidden inside the female during mating. In several bushcricket species, males bear a pair of sclerotized genital appendices called titillators. By employing synchrotron-based *in vivo* X-ray cineradiography on mating couples, we were able to visualize titillator movement and spermatophore attachment inside the female. Titillators are inserted and retracted rhythmically. During insertion the titillator processes tap the soft and sensillae-covered dorsal side of the female’s flap-like genital fold, which covers the opening of the female’s genitalia, without tissue penetration. Titillators thus appear to be initially used for stimulation; later they may apply pressure that forces the female’s genital fold to stay open, thereby aiding mechanically in spermatophore transfer.

The evolution of animal genital structures is widely held to be driven by sexual selection^1^,^2^,^3^,^4^. It is not surprising, therefore, that most male genitalia are evolving much faster than other body parts^5^,^6^. These organs are extremely diverse and can be used at different stages during mating^5^. In several insect species, male claws, hooks, spikes or barbs are relevant for sperm transfer^4^. Additionally, some males’ genital organs are also proposed to act as stimulators, motivating the females to accept the transfer of the sperm or have further post copulatory effects on sperm storage or re-mating^1^,^7^. Males of numerous insect species bear elaborate devices to mechanically connect copulating pairs^8^,^9^,^10^.

In bushcrickets (Ensifera: Tettigoniidae), males possess a pair of claw-like cerci - grasping organs that support the connection with the female^11^ and, in some species, control mating duration^12^. In several Tettigoniidae subfamilies the male’s phallus, covering the distal end of the ejaculatory duct, bears additional sclerotized genital structures, the titillators^13^, which are usually concealed inside the male’s genital chamber (see Fig. 1 and the Supplementary Material). During copulation, they are rhythmically inserted inside the female’s genital chamber^14^,^15^. Across bushcricket species, titillators vary highly in quantity, structure, shape and number of spines^13^,^16^. A comparative phylogenetic analysis of 54 Tettigoniid species showed that copulations are shorter in subfamilies without titillators in comparison to subfamilies with titillators^16^. Additionally, copulation duration in species with complex titillators was positively correlated with the weight of the spermatophore, which contains the male’s sperm and is transferred at the end of the mating.

Males of the middle European bushcricket *Metrioptera roeselii* (Hagenbach, 1822) possess one pair of sclerotized titillators with processes strongly projecting into the genital chamber^14^ (Fig. S2). *M. roeselii* is a polyandrous species and females are known to mate up to five times during their lifetime^17^. Mating of this species takes place in three steps: (a) approach, (b) copulation and (c) spermatophore transfer^15^. The female approaches her mating partner phonotactically, following his courtship song. Once the female reaches the stridulating male, she mounts the male’s back and the male establishes the mating position by grasping the female with his cerci (Fig. 1). This behavior is standard practice for Tettigoniids. During copulation, the male inserts his titillators rhythmically into the female’s genitalia between seven and 18 times per minute, tapping a region of the female’s genital fold, which is densely covered with sensory cells^14^. This flap-like structure on the end of the female’s abdomen covers the genital chamber and is opened during the copulation. After approximately 30 minutes the male ejects a large spermatophore, which he attaches to the female’s genital opening. The spermatophore consists of the ampulla containing the sperm, the ejaculate, and a large spermatophylax^18^. Directly after the mating, the females begin to feed on this proteinaceous nuptial gift, beginning with the spermatophylax. During this time, the sperm migrates from the ampullae into the female’s spermatheca^19^. If the spermatophore transfer is not adequately ensured, this migration cannot take place and the sperm will otherwise be consumed by the female, thus impairing the reproductive success of the donor of the nuptial gift. Polygamous bushcricket females use several indicators, such as mating calls and male body mass, to assess the male’s fitness^18^,^20^. In some species, females even reject undesired partners after the beginning of the copulation^19^, suggesting that mate assessment by the female continues through the copulation. Experimental shortening of titillators in *M. roeselii* reduced the male’s capacity to transfer the spermatophore and increased female resistance behaviours during copulation^15^. Therefore, the titillators in this species are expected to be important in spermatophore attachment and/or for suppressing female resistance behaviour via stimulation^14^,^15^. The function of the bushcricket titillators has yet not been completely understood because previous studies could not probe internal dynamics. μCT reconstructions showed only one position of the inserted titillator as the couples were flash-frozen during copulation^14^. Video recordings of mating pairs during the phase of copulation are also not conclusive as the titillators are covered by the female’s and male’s genital tissues for most of their motion sequence^14^.

The aim of this study is to understand the complex function of the male’s titillators in copulation and spermatophore transfer. Using synchrotron-based *in vivo* X-ray cineradiography^21^,^22^,^23^,^24^, we followed the real-time genital movements of mating couples.

## Results

**Figure 2:**
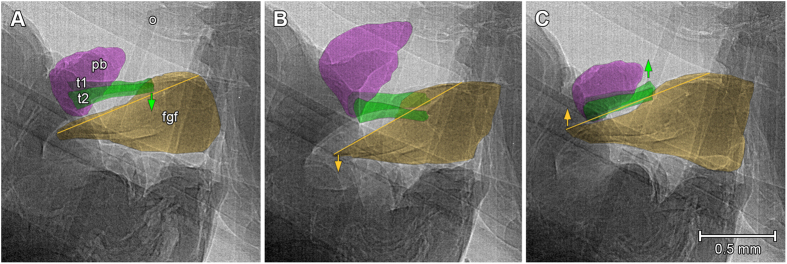
Rhythmic titillator movements of the male (left) open the female’s genital fold (fgf, on the right), revealed by X-ray imaging. The images were extracted from the video, which was recorded at 50 fps, 3.6x magnifications, and 3.06 μm pixel size. The external parts of the titillators are colored in green, the titillator basis is concealed within the male’s abdominal tissues. (**A**) The male’s titillators (t1 and t2, green) contact the female’s genital fold (fgf, yellow, the yellow line indicates the aperture of the fgf) and begin to push it down (green arrow). (**B**) The female’s genital fold opens wider (yellow arrow, the slope of the yellow line becomes steeper), while the titillators push it downwards. In the meantime, the male’s phallobasis (pb, purple) is touching the female’s ovipositor (o). (**C**) The titillators are retracted (green arrow) and the female’s genital fold is moving upwards in its initial position (yellow arrow, the slope of the yellow line levels out).

**Figure 3:**
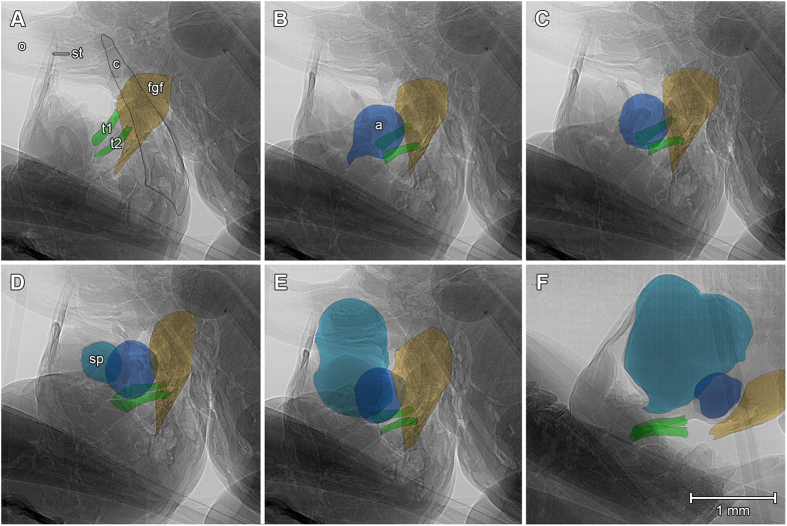
Radiography of male and female genital interaction during spermatophore transfer by a bushcricket mating couple. The images were extracted from the video S2, which was recorded at recorded at 25 fps, 3.6x magnifications, 3.06 μm pixel size. (**A**) During the spermatophore ejection the male’s cerci (c) help to keep the female’s and male’s genitalia in close contact. (**B,C**) The male’s titillators (t1 and t2, green) are pushed down on the female’s genital fold (fgf, yellow) during the ejection of the ampullae (a, dark blue). (**D–F**) While pumping out the spermatophylax (sp, light blue), the ampullae slide over the titillators and are attached on the female’s genital opening.

The scans reveal a rhythmic, symmetrical and synchronous movement pattern of the titillators and the surrounding tissues of the phallobasis. During copulation, both titillator processes are moved from the male’s genital chamber into the female’s genitalia. Once inserted, the titillators tap several times on the middle part of the dorsal side of the female’s genital fold. They are then completely retracted and again pushed into the female’s genital chamber (Video S1). The titillators do not contact the female’s tissues for longer periods, nor do they penetrate it. However, each of these short taps is followed by a wide opening of the female’s flap-like genital fold (Fig. 2 and Video S1). The lymph-filled soft phallobasis is moved together simultaneously with the titillators and contacts the basis of the female’s ovipositor (Fig. 2B and Video S1). The firm grip of the cerci allows the male to hold both genitalia close enough for the copulation movements of the titillators and later to attach the spermatophore onto the female’s genital opening. The tips of the female’s genital fold are at that time placed within the male’s genital chamber between the male’s titillators and his cerci (Fig. 2). The styli at the end of the male’s subgenital plate are placed around the female’s ovipositor; they seem to have no function in copulation and spermatophore transfer (Fig. 3 and Video S2). At the end of the mating, the male ejects a large spermatophore, starting with the ampulla and finishing with the huge proteinaceous spermatophylax (Fig. 3 and Video S2). The spermatophore is released ventrally from the titillators; while it is pumped out, the ampullae press down the titillators on the female’s genital fold, keeping the female’s genitalia wide open to secure spermatophore placement (Video S2).

## Discussion

The live videos show the three major functions of the titillators of *M. roeselii:* the titillators tapping on the membranous dorsal parts of the genital fold stimulate the females, open their genital fold completely and allow the male to transfer the spermatophore. The repeated taps of the titillators on the dorsal part of the female’s genital fold are most likely stimulating the dense field of mechanoreceptors present on that surface^14^. The need to stimulate the female before spermatophore attachment could be a reason why the males insert their titillators not just once, but instead retract and reinsert them up to 18 times per minute during the complete copulation – which can last more than half an hour^15^. In *M. roeselii*, females which mated with partners possessing manipulatively asymmetrically shortened titillators (one titillator shortened, one normal length) displayed conspicuous resistance behaviour during copulation^15^. Some females showing resistance additionally kept their genital fold closed during copulation. The unsatisfactory titillator stimulation of these manipulated males may lead the females to disturb the copulation and try to prevent spermatophore transfer by keeping the genital fold closed. The titillators do therefore function as contact courtship devices during copulation.

The titillators are apparently important for the correct attachment of the spermatophore^15^. On one hand this might result from a female’s cooperation due to anticipated stimulation by the titillators, on the other hand titillators aid in holding the female’s subgenital plate open: the positioning of the female’s genital fold in between the male’s titillators and the cerci basal part prevent the female from closing it. The complete opening of the female’s genital fold is necessary for a proper spermatophore attachment. This might be a reason for the inability of males with both titillators manipulatively shortened to transfer their spermatophore correctly to the females^15^. Therefore, the titillator action assures the reproductive success of the male. Moreover, the spermatophore, which is produced by the accessory glands dorsally from the base of the titillators, slides over the titillators when pumped out, pushing them down on the female’s genital fold (Fig. 3 and Video S2). In this manner, the titillators hold the female’s genital chamber wide open and guide the spermatophore to its attachment point, assuring the deposition of the ampullae next to the opening of the seminal duct.

The rapid evolution of titillators compared to other body parts, as well as the morphological differences between bushcricket species, could be explained by sexual selection. X-ray videos revealed that no structure on the female genital fold fit precisely with the male’s titillators when they tapped. This finding excludes the hypothesis that the male’s organs act as “keys” which bind inside female genital “locks” that function to prevent hybridization. Furthermore, all evidence speaks against forcible use of the titillators. No penetration of the female’s tissues was visible in the X-ray videos, consistent with the previous finding that the titillators do not inflict damage to the females during mating^14^. This excludes any hooking or direct mate securing function of titillators. We therefore hypothesize two scenarios for the evolution of bushcricket titillators. On one hand, titillators may have evolved by sexually antagonistic coevolution of the sexes, allowing males to forcefully hold the female’s genital fold open and thus gain control over the spermatophore attachment. On the other hand, the major evolutionary driving force for titillator evolution could be female choice for males which provide proper genital stimulation. A properly stimulated female keeps her genital fold open and restrains from resistance. This allows the male complete access to her genital chamber for successful spermatophore attachment close to the opening of her reproductive tract. Moreover, female cooperation is required for mating: she has to mount the male’s back before he can achieve the correct position by grasping her with his cerci. Without opening her genital fold at least partly, the male is probably not able to tap on this surface or to open it for spermatophore deposition.

Our live-videos show for the first times both the stimulatory function and the supporting action for spermatophore attachment.

## Materials and Methods

### Study species

Animals were caught as nymphs close to Berlin, Germany (Stahnsdorf 52°23′14″N, 13°12′54″E). They were kept in plastic boxes (30 × 40 × 30 cm) covered with gauze under a temperature regime ranging from 22 to 25 °C and a light-dark cycle of 16:8 h. Animals were fed ad libitum with mixtures of fresh grass, oat flakes, fish food pellets (Tetramin®) and bee pollen. Water was sprinkled three to five times a day on the side walls of the boxes. All experiments were performed at room temperature (21–23 °C).

### Preparation of animals for *in vivo* radiography

Randomly chosen virgin males and females were paired at the ages of nine to twelve days. We needed to immobilize all the other extremities and the body of the bushcrickets, to focus on the interaction of the male’s titillators with the female’s genitalia. Couples were placed on the specimen holder immediately after beginning of the copulation. The thorax and abdomen of the male were attached with adhesive tape on the specimen holder. Liquid glue was used to fixate the female and the extremities of the male. The immobilized couples were then integrated into the experimental setup (Fig. S1) and euthanized after the scans.

### *In vivo* X-ray cineradiography

The scans were performed at the TOPO-TOMO beamline^25^,^26^ of the ANKA Synchrotron Radiation Facility, using a filtered parallel polychromatic X-ray beam at a spectrum peak of about 15 keV. An indirect detector system composed of a 12 μm LSO: Tb scintillator^27^, diffraction limited optical microscope (Optique Peter^28^) and 12 bit pco. dimax high speed camera with 2016 × 2016 pixels resolution^29^ was employed to capture the radiographic sequences. All data from these experiments are maintained on a public server. More than 40 couples were scanned, resulting in four good quality videos showing the genitalia during copulation and one showing the spermatophore transfer.

### Morphology of male titillators

For morphological examination of the titillators, we used four male bushcrickets fixed in 70% Ethanol. The caudal end of the male was macerated in 10% KOH for four hours at 80 °C to eliminate all mesodermal and ectodermal tissues and gain full visibility on the titillators and accessory glands. In the view from ventral the cerci were removed with small scissors to gain unrestricted visibility. Photos were taken with a digital microscope (Leica M205C) and a Leica DFC420 digital camera.

## Additional Information

**How to cite this article**: Wulff, N. C. *et al*. Copulatory courtship by internal genitalia in bushcrickets. *Sci. Rep.* **7**, 42345; doi: 10.1038/srep42345 (2017).

**Publisher's note:** Springer Nature remains neutral with regard to jurisdictional claims in published maps and institutional affiliations.

## Supplementary Material

Supplementary Material

Supplementary Movie S1

Supplementary Movie S2

## Figures and Tables

**Figure 1:**
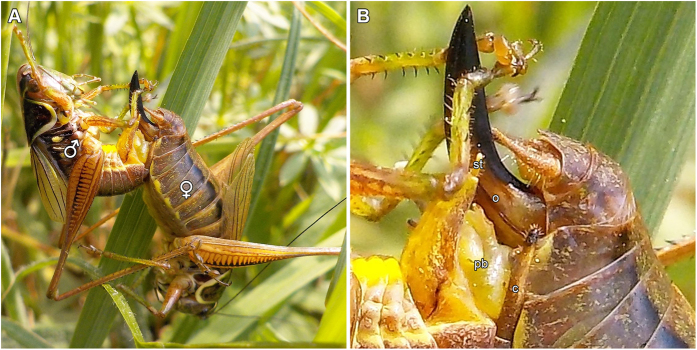
Bushcricket mating position. (**A**) Mating couple of *M. roeselii* during copulation. (**B**) Enlargement of the male’s and female’s genitalia. The male (left) grasps the female (right) with its cerci (c) and pulls the genitalia close to each other. The female’s ovipositor (o) is touched by the male’s styli (st) and often also by its first two leg pairs. The female’s genital fold and the male’s titillators are covered by the male’s phallobasis (pb).
