# Supplementary material for: Copulatory courtship by internal genitalia in bushcrickets
Source: Sci Rep. 2017 Feb 7;7:42345. doi: 10.1038/srep42345 (PMC5294567; doi:10.1038/srep42345)
Supplement: Supplementary Material [file srep42345-s1.pdf]

Supplementary Materials for

Copulatory courtship by internal genitalia in bushcrickets

Nadja C. Wulff, Thomas van de Kamp, Tomy dos Santos Rolo, Tilo Baumbach, Gerlind U.C. Lehmann
correspondence to: nadja_wulff@yahoo.de

This PDF file includes:

SupplementaryText
Figs. S1 to S2
Captions for Movies S1 to S2

Other Supplementary Materials for this manuscript includes the following:

Movies S1 to S2

Supplementary Text

Morphology of male titillators

The male's phallus and the titillator base are placed inside the soft genital chamber, covered dorsally by the styli at the end of the subgenital plate (Fig.S2C) and ventrally by the claw-like cerci (Fig. S2B). The titillator processes are surrounded by the two lymph-filled sacks of the phallobasis. The accessory glands, which produce components of the spermatophylax, lie dorsally from the dark-brown-coloured titillator base without direct physical connection (Fig. S2). The ejaculate leaves the accessory glands in two ejaculatory ducts, which lie near the dorsal parts of the abdomen, and is guided out of them dorsally from the titillator processes.

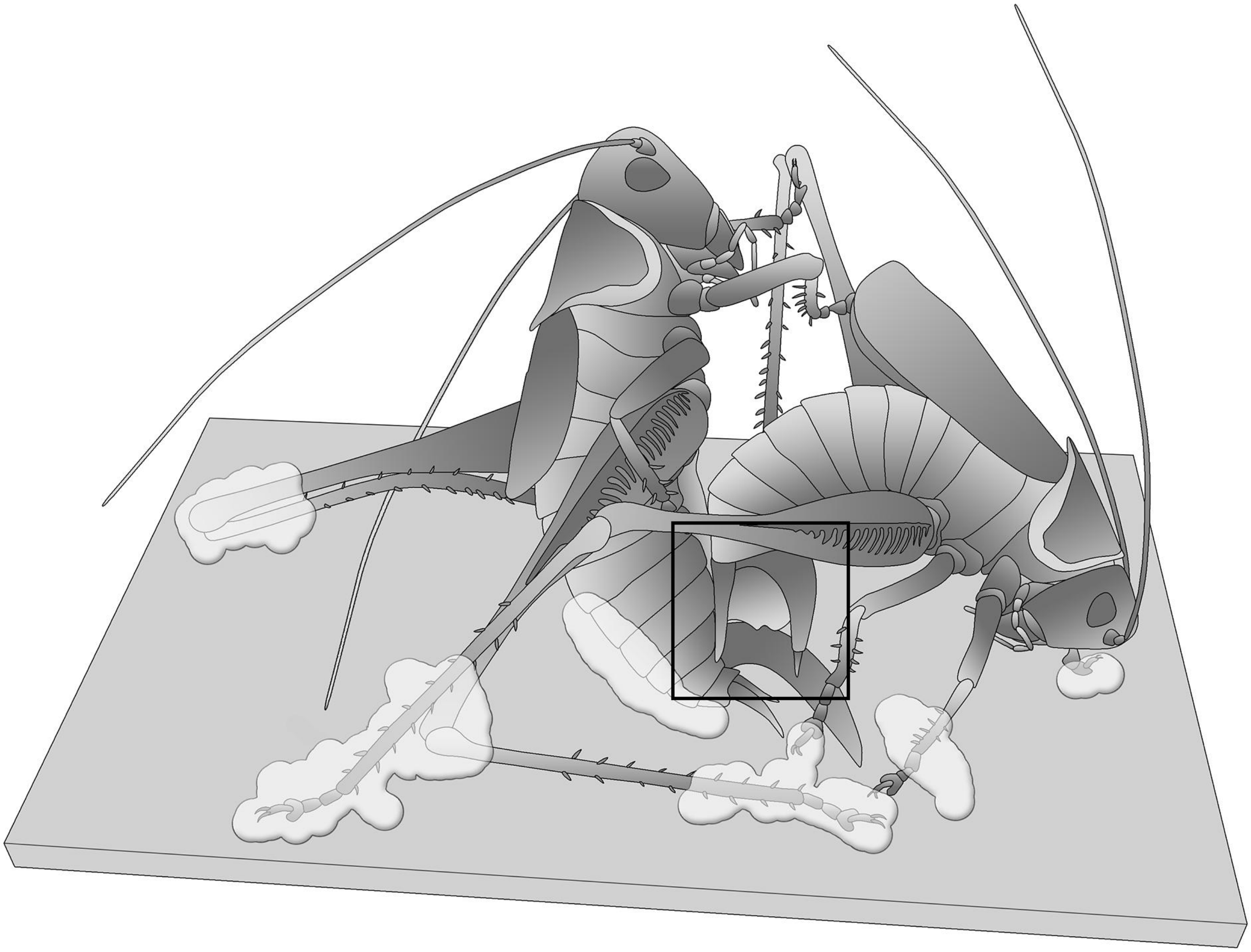

Fig. S1
Bushcricket couple immobilized for in-vivo X-ray cineradiography of male and female genital interaction. Female on the left side, male on the right side. The black square indicates the part of the genitalia which was scanned.

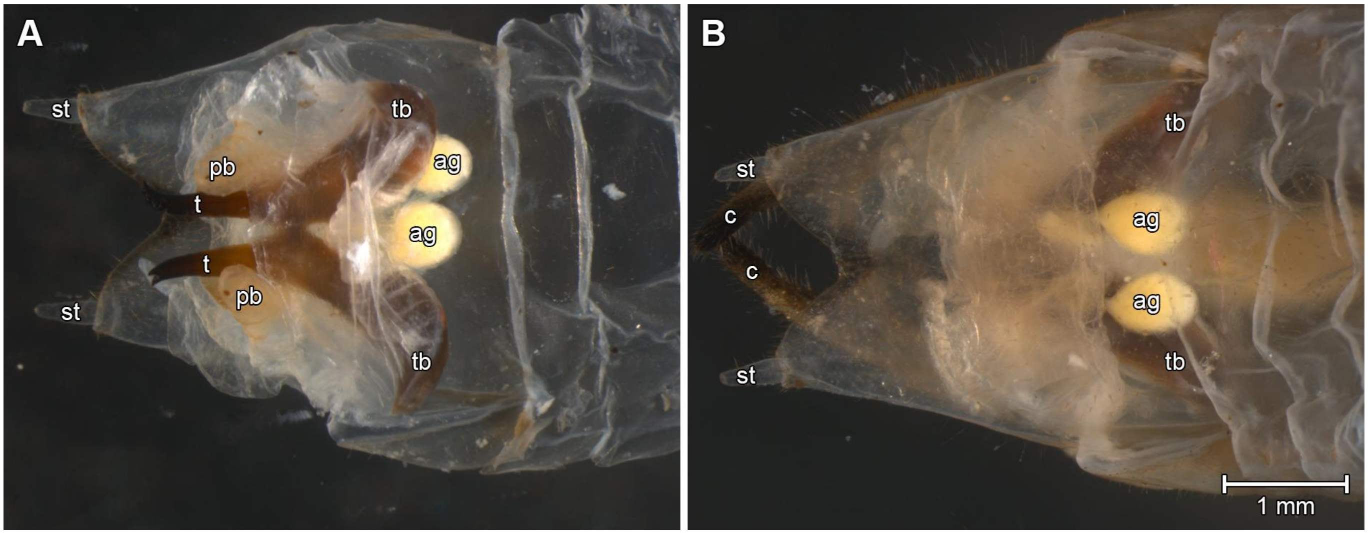

Fig. S2

Caudal end of the abdomen of a *M. roeselii* male macerated in 10% KOH, showing the styli (S), the titillators (T), the phallobasis (PH), the accessory glands (AG), the titillator basis (TB) and the cerci (C). **(A)** View from ventral, cerci removed. **(B)** View from dorsal through the cuticula.

Movie S1

X-ray video of a cineradiography of male and female genital interaction by a bushcricket mating couple during copulation, recorded at 50 fps, 3.6 x magnifications, and 3.06 µm pixel sizes. The titillator basis is concealed within the male's abdominal tissues. The male's titillator tips contact the female's genital fold rhythmically, which opens wider. In the meantime the male's phallobasis is touching the female's ovipositor. Only when the titillators are retracted, the female's genital fold is moving upwards in its initial position.

Movie S2

X-ray video of a cineradiography of male and female genital interaction during spermatophore transfer by a bushcricket mating couple, recorded at 25 fps, 3.6 x magnifications, and 3.06 µm pixel sizes. During the ejection of the ampullae, the male's titillators are pushed down on the female's genital fold. While pumping out the spermatophylax, the ampullae slide over the titillators and are attached on the female's genital opening.
